# FOXP3/HAT1 Axis Controls Treg Infiltration in the Tumor Microenvironment by Inducing CCR4 Expression in Breast Cancer

**DOI:** 10.3389/fimmu.2022.740588

**Published:** 2022-02-09

**Authors:** Tania Sarkar, Subhanki Dhar, Dwaipayan Chakraborty, Subhadip Pati, Sayantan Bose, Abir K. Panda, Udit Basak, Sourio Chakraborty, Sumon Mukherjee, Aharna Guin, Kuladip Jana, Diptendra K. Sarkar, Gaurisankar Sa

**Affiliations:** ^1^ Division of Molecular Medicine, Bose Institute, Kolkata, India; ^2^ Department of Surgery, Institute of Post Graduate Medical Education & Research-Seth Sukhlal Karnani Memorial Hospital (IPGMER-SSKM) Hospital, Kolkata, India

**Keywords:** Treg cells, FOXP3, CCR4, Treg infiltration, tumor microenvironment (TME)

## Abstract

Infiltrating T-regulatory cells in the tumor microenvironment is a key impediment to immunotherapy and is linked to a poor prognosis. We found that tumor-infiltrating Tregs express a higher expression of the chemokine receptor CCR4 than peripheral Tregs in breast cancer patients. CCL22 and CCL17 are released by tumor cells and tumor-associated macrophages, attracting CCR4^+^ Tregs to the tumor site. The Treg lineage-specific transcription factor FOXP3 changes the *CCR4* promoter epigenetically in conjunction with HAT1 to provide a space for FOXP3 binding and activation of the CCR4 gene. To increase CCR4 expression in Tregs, the FOXP3/HAT1 axis is required for permissive (K23 and K27) or repressive (K14 and K18) acetylation of histone-3. In murine breast and melanoma tumor models, genetic ablation of FOXP3 reduced CCR4^+^ Treg infiltration and tumor size while also restoring anti-tumor immunity. Overexpression of FOXP3, on the other hand, increased CCR4^+^ Treg infiltration, resulting in a decreased anti-tumor immune response and tumor progression. These findings point to FOXP3 playing a new role in the tumor microenvironment as a transcriptional activator of CCR4 and a regulator of Treg infiltration.

## Introduction

Tumor growth and development is a complex and dynamic process. Tumor microenvironment (TME), which is made up of a growing tumor mass, extracellular matrix, immune and stromal cells, and cell-secreted cytokines and chemokines, aids carcinogenesis (1). Growing tumor mass alters the immune system, resulting in a tolerogenic environment in the TME (2, 3). The development of T-regulatory (Treg) cells is the most important and meaningful change among them. Among the diverse immune cells, Treg cells are critical components that play a major role in tumor immunological escape (4). Treg cells promote peripheral tolerance on the one hand, but their effects on tumor immunosurveillance are damaging on the other.

Tregs can arise spontaneously or in pathological conditions such as cancer from naïve or activated T cells. Natural Tregs (nTregs) develop in the thymus by stimulation of self-antigens (5). Tregs can also develop from naïve T cells during specific stimulation in the peripheral circulation (6). These Tregs are known as peripheral Tregs (pTregs) or induced Tregs (iTregs). iTreg cells phenotypically resemble nTregs (7). Tregs suppress the immune response in a variety of ways (8, 9). FOXP3 (fork-head F-box protein), an X-chromosome-encoded lineage-specific transcription factor, is necessary for Treg cells’ suppressive functions (10, 11). FOXP3 activates or represses the transcription of its target genes to achieve its numerous tasks (12, 13). TGFβ is a key regulator of the signaling pathways that lead to Treg development by initiating and maintaining FOXP3 expression in CD4^+^CD25^-^ precursors (14). During tumor progression, Treg expansion decreases the function of tumor-specific T-effector (Teff) cells (12, 15, 16). Tregs are detected in high numbers in the peripheral blood and tumor tissue of patients, and a high number of Tregs is connected to a poor prognosis (13, 17).

Tregs, according to studies, infiltrate the tumor site from the peripheral circulation, creating an environment conducive to tumor progression; thus, they represent a key stumbling block in cancer treatment (18). Tregs decrease Teff cell and NK cell responses in cancer, interfering with both acquired and innate immunity (19). Tregs have a multitude of chemokine receptors that respond to chemokines released by the tumor mass as it grows. The interaction of chemokines and chemokine receptors on cell surfaces is required for its migration (20). As a result of chemokines generated by tumor cells, a concentration gradient is created, which Tregs follow to travel to the tumor site (21–23). The importance of the chemokine network in cancer is becoming increasingly clear. Treg compartmentalization and trafficking are tissue- and organ-specific, according to studies, and unique chemokine receptor expression leads to Treg selective retention and trafficking at regulatory locations. Tregs express the chemokine receptor CCR2 and interact with the ligand CCL2 as they move towards inflammatory tissue. CCR7–CCL19 interaction is also required for Treg trafficking to lymph nodes (24). Tregs, on the other hand, express a specific chemokine receptor, CCR4 (CD194), during metastasis (25). CCR4 is a chemokine receptor that Th2 cells, Treg cells, and skin-homing effector/memory T cells all express in different ways (26). CCR4, which binds to macrophage-derived chemokine (MDC/CCL22) and thymus- and activation-regulated chemokine (TARC/CCL17), is predominantly expressed by tumor-associated Treg cells (27, 28).

Both CCL22 and CCL17, which are produced by tumor cells, tumor-associated macrophages, and dendritic cells, establish a concentration gradient around the tumor mass and attract CCR4^+^ Tregs to maintain immunological homeostasis in the tumor vicinity (29–31). When CCL22/CCL17 interacts with CCR4, a signaling cascade occurs, causing changes in cell shape and increased motility in Tregs, allowing them to infiltrate the TME (32). These tumor Tregs are functionally suppressive, capable of inhibiting tumor-specific immunity, promoting tumor development, and predicting poor prognosis (33). The importance of Treg tumor infiltration is well known, as Tregs make the TME tolerogenic and also reduce the effectiveness of anti-cancer therapy (34). The use of a humanized anti-CCR4 antibody (mogamulizumab) for the treatment of adult T cell leukemia and cutaneous T-cell lymphoma has recently been licensed, highlighting the importance of CCR4 in tumor growth (35, 36). However, little is known regarding *CCR4* transcriptional regulation in Treg cells in breast cancer. Our current research focuses on CCR4 modulation in Tregs and its implications for breast cancer.

Our research identifies a FOXP3^+^ Treg population in tumor-infiltrating CD4^+^ lymphocytes that are largely CCR4^+^. The FOXP3^+^CCR4^+^ Tregs from breast cancer patients’ peripheral circulation infiltrate tumor site in response to TME-secreted CCL17 and CCL22. The lineage-specific transcription factor FOXP3 controls its target genes in Treg cells (9), which led to the hypothesis that FOXP3 might also operate as a transcriptional regulator of CCR4. Genome-wide analysis of FOXP3-binding sites revealed that FOXP3 can act both as a transcriptional activator and as a repressor (37). Our predicted docking module suggests that the *CCR4* promoter has a FOXP3 responsive element. FOXP3 and HAT1 (Histone acetyl transferase-1) were shown to modify the *CCR4* promoter epigenetically to create a space for FOXP3 binding to transcriptionally activate the *CCR4* gene. Interference with FOXP3 binding to the *CCR4* promoter reduces Treg infiltration in the tumor site, reactivating the suppressed anti-tumor immune response. Understanding *CCR4’s* transcriptional regulation will lead to novel approaches to prevent Treg infiltration in tumors. According to current research, it will soon be discovered as a technique to improve the immune system’s response to tumors.

## Materials and Methods

### Patients

The participants in this study were 23 female breast cancer patients (Stage I, *n* = 6; Stage II, *n* = 8; Stage III and IV, *n* = 9; ages: 18–65 years) and 10 age-/sex-matched healthy people (**Table S1**). The tumor stage was assessed using the TNM classification of the International Union Against Cancer from 2002. Three patients with Stage II breast cancer who had mastectomy were given ipsilateral normal breast tissues. The ethics committees of the ESI Post-Graduate Institute of Medical Science and Research, Kolkata (Approval No: ESI-PGIMSR/MKT/IEC/13/2017 dated December 22, 2017), the Institute of Post-Graduate Medical Education and Research Oversight Committee (Approval No. IPGME/IEC/13/2017 dated December 22, 2017), and Human Ethics Committee, Bose Institute (Approval No: BIHEC/2017-18/7, dated May 28, 2017) approved the collection of post-operative breast tumor tissue samples and peripheral blood from breast cancer patients/normal. All patients enrolled in the trial gave their informed consent.

### T Cell Isolation and Culture

To obtain PBMC, peripheral blood was centrifuged at 800×*g* for 45 min over the lymphocyte separation medium (Histopaque). For T-cell polarization, magnetic-bead sorted naïve CD4^+^CD45RA^+^ T cells from cord blood were activated with anti-CD3 and anti-CD28 beads (Invitrogen) before being allowed to develop into Tregs in the presence of recombinant TGFβ (5 ng/ml) and IL2 (50 U/ml) and anti-IFNγ antibody (PeproTech). Flow cytometry was used to determine the purity of the enhanced cells, which was consistently >90%. During flow cytometric analysis, LymphoGate was employed to detect lymphocytes. Treg differentiation was confirmed by flow cytometry and qPCR. Cells were cultured in a complete RPMI-1640 medium supplemented with 10% FBS at 37°C in a humidified 5% CO_2_ incubator. All experiments were performed with mycoplasma-free cells.

### THP1 Culture

THP1 (CVCL 0006), a well-established monocyte cell line, was grown in RPMI-1640 with 10% serum. Monocytes were treated with PMA (5 ng/ml; Sigma) to generate the M-M0 subtype. THP1 can be polarized from Mϕ-M0 to Mϕ-M1 by adding LPS (100 ng/ml; Peprotech) and IFNγ (20 ng/ml; PeproTech) to it. Mϕ-M0 was also polarized to the Mϕ-M2 subtype by treating it with IL4 (20 ng/ml; PeproTech) for 48 h. The cell line has been authenticated using STR (or SNP) profiling within the last 3 years by the National Centre for Cell Science, Pune, India. All experiments were performed on mycoplasma-free cells.

### *Ex Vivo* Tumor Microenvironment

To generate *ex vivo* TME, tumor tissues were collected from breast cancer patients undergoing surgical procedures, minced, and stirred in cell dissociation reagent containing collagenase Type IV in DMEM/F-12 for 2 h. The tumor cell suspension was filtered through nylon mesh and centrifuged for 10 min at 250×*g*. To make a single-cell suspension, the cell pellet was washed with serum-free RPMI 1640 and resuspended in complete RPMI 1640 media. CD24 and ESA positivity or CD4-/CD8- and CD25 negativity were used to assess the purity of those cells flow cytometrically. T cells from healthy volunteers were over-layered on top of primary breast tumor cells (to mimic the TME *in vitro*) and cultured for 72 h to generate Tregs *ex vivo* (**Figure S1**) (38).

### Flow Cytometry and Cell Sorting

Fluorophore-conjugated CD4 (FITC/-PE), CD25 (PE/PE-CY7), CD127 (AF-647), CTLA4 (PE), FOXP3 (AF-647), and CCR4 (BV-421/PE) antibodies (Biolegend/BD Bioscience) were used to stain cells for phenotyping. The expression of Th1- and Th2-specific transcription factors T-bet (Santa Cruz) and GATA3 (Santa Cruz) was used to determine the percentage of Th1 and Th2 cells, respectively. Cells were stimulated for 5 h at 37°C with cell activation cocktail and brefeldin-A (Biolegend) before being stained with anti-IL10 (BV-421) using the Cytofix/CytoPerm Plus kit (BD Biosciences). The fluorescent-conjugated annexin-V was used to assess cellular apoptosis (BD Bioscience). For the purification of naïve and Treg cells, CD4^+^ cells, labeled for CD45RA, CD25, and CTLA4, were subjected to high-speed cell sorting (FACS-ARIA, BD Biosciences) to obtain CD25^+^CTLA4^+^ Treg and CD45RA^+^ naïve T cells. Data were acquired and analyzed by FACS using FACS-Suite and FlowJo software (BD Biosciences). Quadrants were constructed based on signals utilizing FMO and unstained controls to quantify stained cells in contour plots. For t-SNE analysis, the lymphocyte population was gated and the CD4^+^ population was subsequently marked. The lymphocyte population from blood acquired from a breast cancer patient in the cohort was a down-sample with 5,000 cells, and the dimensionality reduction was done with the population to create t-SNE parameters.

### Transduction

*Ex vivo*-generated Tregs (2 × 10^6^ cells) were transduced with FOXP3-/CCR4-shRNA (Thermo-Fisher Scientific), FOXP3-/CCR4-cDNA (ADDGENE), or control vector by electroporation (Invitrogen, USA) in a single-pulsed method (voltage 260 V and capacitance 1050 μF). Transduced cells were cultured for 48 h before being exposed to downstream tests. We routinely achieved a transduction effectiveness of 85%–90% under these conditions without compromising cell viability.

### cDNA Preparation and Real-Time PCR

Treg cells (2 × 10^6^ cells) were stimulated with PMA and ionomycin for 4 h at 37°C before RNA was isolated using TRIzol reagent (Thermo Fisher Scientific) and reverse transcribed using Verso-cDNA synthesis kit (Thermo-Fisher Scientific). FastStart Essential DNA Green Master (Roche) was used for qPCR analysis, which was carried out using a GeneAmp PCR-2720 (Applied Biosystems, CA, USA). Melting curves using LC96 SW1.1 software were used to assess amplification products using SYBR-green detection (Roche). A 2^–ΔΔCt^ approach was used to analyze the data. The expression level of the house-keeping gene, GAPDH, was used to normalize for variations in the amount of RNA. The respective primers list is given in **Table S2**.

### ChIP and Re-ChIP

To cross-link the protein–DNA complexes, *ex vivo*-generated Tregs (2 × 10^6^ cells) were cross-linked in 1% *p*-formaldehyde for 15 min at 37°C. Soluble chromatin (7–20 μg) was treated with 4–5 μg of antibody overnight at 4°C after being sonicated and pre-cleared with protein-A agarose/salmon-sperm DNA. FOXP3, RNA POL-II (Santa Cruz), HAT1, acetylated-H3 (K14, K18, K23, and K27), anti-rabbit, and anti-mouse immunoglobulin-G antibodies (Cell Signaling Technology) were used for ChIP and Re-ChIP assays. In addition, a negative control IgG (Sigma-Aldrich) was employed. Following immunoprecipitation and washing, DNA from each immunoprecipitation was measured using distinct primer sets (**Table S2**) for the FOXP3 and RNA POL-II binding sites on the *CCR4* promoter by qPCR. After eliminating background immunoprecipitation assessed with nonspecific IgG or the input sample, the fold-change in occupancy of each protein at the *CCR4*-promoter region of Treg relative to the T naïve cell was estimated. DNA–protein complexes immunoprecipitated with anti-FOXP3 antibody in primary ChIP were eluted with 25 μl of 10 mM dithiothreitol for 30 min at 37°C and diluted 20 times with Re-ChIP buffer for the Re-ChIP tests (1% Triton X-100, 2 mM EDTA, 150 mM NaCl, and 20 mM Tris-HCl; pH 7.5). After that, the complexes were re-immunoprecipitated with anti-HAT1 antibody before qPCR examination of the precipitated chromatin DNA (39).

### Confocal Microscopy

Breast tumor patient-derived Tregs and naïve T cells were washed in PBS with 1% BSA. Then 1 × 10^4^ cells were allowed to adhere to poly-L-lysine-coated slides. Cells were fixed with 4% *p*-formaldehyde and permeabilized with 0.2% saponin. After blocking with 3% BSA, cells were incubated with anti-FOXP3/-CCR4 (rabbit/mouse mAb), followed by fluorescence-tagged secondary antibodies (Invitrogen) and DAPI (BD Biosciences). Cells were visualized in Leica confocal microscope, with DPX mounting medium, at 60× magnification. Images were analyzed by ImageJ software (40).

### Trans-Well Assay

Polycarbonate filters (pore size, 5 µm; Cell Culture Inserts, Corning) were used for Trans-well assay that helps direct the passage of the cells from the upper to the lower chamber. The upper chamber was plated with RPMI-1640 medium containing patient-derived Tregs and the lower chamber was plated with tumor cell supernatant containing recombinant CCL22 (PeproTech). After 4 h at 37°C, cells migrated at the lower chamber were collected, immunostained, and analyzed in a flow cytometer. To check the role of FOXP3 in Tregs migration, *ex vivo*-generated Tregs were first transfected with lentivirus containing desired clones and the transfected cells were placed in the upper chamber of trans-well plates.

### Western Blot

Cells were homogenized in buffer (20 mM Hepes, pH 7.5, 10 mM KCl, 1.5 mM MgCl_2_, 1 mM Na-EDTA, 1 mM EGTA, and 1 mM DTT) supplemented with protease and phosphatase cocktail inhibitor. A total of 60 μg of protein was resolved using 10% SDS-PAGE, transferred to nitrocellulose membrane, and probed with specific antibodies CCR4 (Santa Cruz) and FOXP3 (Santa Cruz). Secondary antibodies used (1:10,000) were conjugated with HRP (Sigma). After that, the immunoblots were developed by chemiluminescence method using luminol and coumaric acid. Equal protein loading was confirmed with anti-β-actin antibody (mAb) (Santa Cruz).

### Lentivirus Preparation

Packaging of lentiviruses was performed by transient transfection of HEK cells. One day before transfection, HEK cells were seeded in a T75 flask at 1 × 10^5^ cells/cm^2^ in DMEM supplemented with 10% FCS, 1 mM pyruvate, and 40 mg/ml gentamicin. The calcium phosphate precipitation method was used to co-transfect cells using 7.5 μg of gag/pol packaging plasmid psPAX2, 7.5 μg of pGIPZ-Foxp3-shRNA/Foxp3-cDNA transfer vector, and 4 μg of envelope plasmid pMD2.G using the Profection mammalian transfection kit (Promega, USA). Transfections were carried out in 10 ml of DMEM without antibiotics, and the cells were grown for 16 h. After that, the medium was replaced with complete DMEM, and after 48 h, the supernatant was collected. Centrifugation at 1,500 rpm for 5 min at 4°C was used to remove cell debris, which was then passed through a 0.45-μm pore PES filter. For concentration, 20 ml of supernatant was centrifuged at 4,000 rpm for 20 min at 4°C in a 12% polyethylene glycol (PEG-8000) solution. In serum and antibiotic-free DMEM, pelted viruses were re-suspended and the aliquots were kept at −80°C for further use (41).

### Animal Model

BALB/c and C57/BL6 mice weighing 20–25 g (in-house animal facility) were kept in a temperature-controlled environment with a light–dark cycle. All animal experiments were carried out following the National Institutes of Health’s Principles of Laboratory Animal Care (NIH publication No. 85-23, revised in 1985) as well as Indian laws on “Animal Protection” under the provision of the Bose Institute Animal Ethics Committee for the control and supervision of animal experiments (Reg. No. 95/99/CPCSEA; Approval No: IAEC/BI/47/2017). A total of 2 × 10^6^ isogenic mammary carcinoma 4T1 or B16/F10 melanoma cells (ATCC) were injected in the breast fat pad of BALB/c mice or subcutaneously in C57/BL6 mice, respectively. Mice were separated into five groups, each with three mice: (i) control, (ii) tumor alone (no transduction), (iii) control vector, (iv) Foxp3-shRNA, and (v) Foxp3-cDNA. The experiments were repeated twice. Lentivirus-bearing desired clones were injected into the tail vein after 7 days of tumor inoculation. Mice were tracked for the next 21 days. Every other day, the tumor volume was measured. The tumor volume was calculated using the formula V = (W2 × L)/2 (caliper measurements), where V represents the tumor volume, W represents the tumor width, and L represents the tumor length. After 21 days, the mice were sacrificed, and Tregs from PBMC and the tumor site location were examined using flow cytometry (42).

### Bioinformatics and Statistical Analysis

The correlation study between FOXP3 and CCR4 was executed using R2: Genomics Analysis and Visualization Platform. The same dataset was used to construct t-SNE plots for FOXP3 and CCR4. MatInspector software was used to analyze the *CCR4* promoter. Multiple sequence alignments of *CCR4* promoter between human and mouse was done by Clustal W. Unless otherwise stated, values are shown as the standard error of the mean (SEM). A 2-way ANOVA was used to compare different experimental groups, followed by a post-hoc Bonferroni modification of the multiple comparison *t*-test. A Student’s *t*-test was used to examine the significance of the differences between mean values when suitable. At *p* < 0.05, the results were considered significant. Graph Pad software was used to create a heatmap for the fold-change values. Prism software was used to analyze the data (Graph Pad software).

## Results

### Tumor-Infiltrating Treg Cells Showed Higher Expression of CCR4 Than the Peripheral Circulation in Breast Cancer Patients

**Figure 1:**
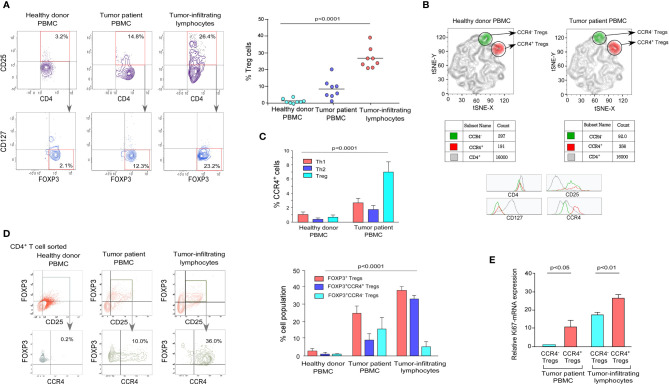
Tumor-infiltrating Treg cells showed higher expression of CCR4 than the peripheral circulation in breast cancer patients. **(A)** Representative flow cytometry data of CD4^+^CD25^+^CD127^-^FOXP3^+^ Tregs from the peripheral circulation of an age-/sex-matched healthy individual, breast cancer patient, and breast tumor tissue (left panel). In a scatter plot (*n* = 8; right panel), percent Treg populations from different cohorts were represented. **(B)** Comparative t-SNE analysis of CCR4^+^ (shown in red/CD4^+^CD25^+^CD127^-^CCR4^+^) and CCR4^-^ (shown in green/CD4^+^CD25^+^CD127^-^CCR4^-^) Tregs within the proportion of CD4^+^ T cells in the peripheral circulation of an age-/sex-matched healthy individual and a breast cancer patient. **(C)** Graphical representation of percent CCR4^+^ cells in Th1, Th2, and Treg subpopulations in the peripheral circulation of age-/sex-matched healthy donors and breast cancer patients. **(D)** Flow cytometric representation of CD4^+^CD25^+^FOXP3^+^CCR4^+^ Tregs in the peripheral circulation of a healthy donor and a breast cancer patient, as well as tumor tissue (left panel). Percentage of CD4^+^CD25^+^FOXP3^+^, CD4^+^CD25^+^FOXP3^+^CCR4^+^, and CD4^+^CD25^+^FOXP3^+^CCR4^-^ Tregs in the peripheral circulation of age-/sex-matched healthy donor and breast cancer patients, as well as tumor tissue (right panel). **(E)** Graphical representation of Ki67-mRNA expression in CCR4^-^ and CCR4^+^ Treg compartments separated from peripheral blood and tumor tissue. As an internal control, GAPDH was used. The values are the mean ± SD of three sets of independent experiments.

We obtained peripheral blood and post-operative breast tumor tissue from a cohort of 8 breast cancer patients undergoing surgery to establish the expression pattern of CCR4 in Tregs. We used a mix of phenotypic markers to further characterize human Tregs and their relationship to tumor progression characteristics. FOXP3 was employed as an internal marker, while CD4, CD25, and CD127 were used as surface markers (33). In comparison to age-/sex-matched healthy donors, patients with advanced breast cancer had higher proportions of CD4^+^CD25^+^CD127^-^FOXP3^+^ Tregs in the peripheral circulation and tumor tissue, as shown in **Figure 1A** (**Figure 1A**; left panel). Based on the cell surface marker expression pattern, we chose CD4^+^CD25^+^CD127^-^ as a suitable set of surface markers to sort Tregs for further characterization. The tumor site had a higher percentage of CD4^+^CD25^+^CD127^-^FOXP3^+^ Tregs than the peripheral circulation of breast cancer patients and age-/sex-matched healthy donors (**Figure 1A**; right panel).

CCR4 is a chemokine receptor that is selectively expressed by Tregs in various pathophysiological situations and plays an important role in lymphocyte organ-specific migration (35, 36). The distribution pattern of CCR4 in tumor-associated CD4^+^CD25^+^CD127^-^ Treg populations in healthy donor and tumor patients was determined using a comparative t-SNE method based on multi-color flow cytometry data (**Figure 1B**). The two populations of Tregs (CCR4^+^ and CCR4^-^) are present in both healthy donors and tumor patients, according to CCR4 expression levels; however, the presence of the CCR4^+^ Treg population is higher than the CCR4^-^ Treg population in tumor patients, which indicates that Treg increases the expression of CCR4 to infiltrate in the TME. We intended to evaluate the status of CCR4 expression in other T-cell subsets (Th1 and Th2) because Tregs show a higher expression of CCR4 in tumor settings. When compared to healthy donors, CCR4 expression is significantly increased by Tregs during tumor condition; however, CCR4 expression is not significantly increased by other T-cell subsets (**Figure 1C**).

We evaluated the expression level of CCR4 in both the peripheral circulation (source) and the tumor site (destination) since it is the tumor-specific migratory marker of Tregs. **Figure 1D** shows that the CCR4^+^ Treg population was found in higher frequency in Tregs obtained from the tumor site (30%) than in Tregs obtained from the tumor patient’s peripheral blood (10%), implying that when the Treg acquires CCR4 expression in the peripheral circulation, it migrates to the tumor site. The graphical representation (**Figure 1D**, right panel) also shows that CCR4^+^ Tregs account for the majority of Tregs in tumor tissue, whereas the percentage in the peripheral circulation of both the healthy donor and the tumor patient is relatively low, indicating that only CCR4^+^ Tregs can infiltrate the tumor site and CCR4^-^ Tregs cannot (**Figure 1D**). In comparison to CCR4^-^ Tregs, CCR4^+^ Tregs from both the peripheral circulation and tumor tissue had greater Ki67 expression. CCR4^+^ Tregs from both the peripheral blood and tumor tissue had greater Ki67 expression than CCR4^-^ Tregs, indicating that CCR4^+^ Tregs are more proliferative (**Figure 1E**). The tumor immune escape is facilitated by the eventual proliferation of these CCR4^+^ Treg cells in tumor tissue.

### Cell Surface Receptor CCR4 Interacts With Chemokine CCL22 and CCL17 to Migrate Treg Cells Into Tumor Microenvironment

**Figure 2:**
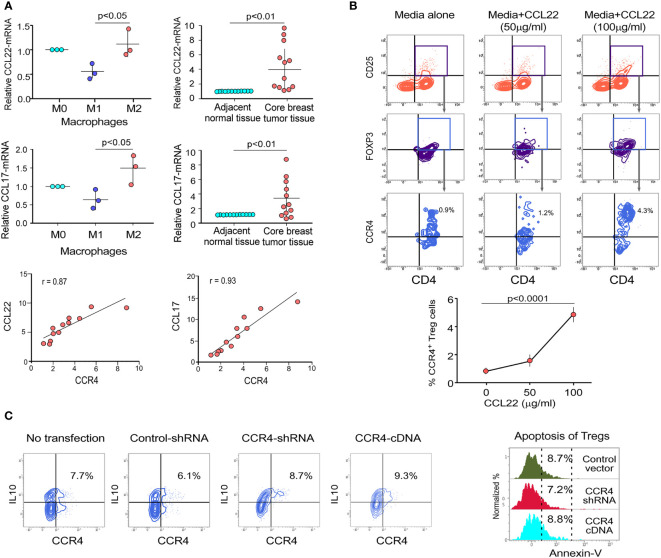
Cell surface receptor CCR4 interacts with chemokine CCL22 and CCL17 to migrate Treg cells into tumor microenvironment. **(A)** Relative mRNA expression of CCL22 (upper left panel) and CCL17 (middle left panel) in various macrophage subpopulations (M0, M1, and M2). CCL22 (upper right panel) and CCL17 (middle right panel) relative mRNA expression in neighboring non-tumor/normal tissue and core breast tumor tissue acquired from breast cancer patients at various stages (*n* = 12). The correlation between CCR4-CCL22 (lower left panel) and CCR4-CCL17 (lower right panel) in core breast tumor tissue taken from various stages of breast cancer patients (*n* = 12) is depicted in representative correlation coefficient plots. **(B)** Flow cytometry was used to examine the migration of CD4^+^CD25^+^FOXP3^+^CCR4^+^ Tregs in response to recombinant CCL22 in a trans-well plate (representative plot) (upper panel). Graphical representation of the percentage of CCR4^+^Treg populations after trans-well migration (lower panel). **(C)** Control-shRNA, CCR4-shRNA, and CCR4-cDNA clones were transfected into *ex vivo*-generated Treg cells, and intracellular IL10 was measured by flow cytometry (left panel). CCR4 expression was genetically altered in *ex vivo*-generated Tregs, and percentage cell death was assessed flow cytometrically by Annexin V positivity after 48 h and depicted in a histo-plot (right panel). As an internal control, GAPDH was used. The values are the mean ± SD of three sets of independent experiments.

CCR4 is a chemokine receptor that responds to CCL22 and CCL17 ligands (27, 28). The interaction between CCL22/CCL17 and CCR4 triggers a signaling cascade in Tregs that results in changes in cell shape and increased motility. In our experiments, we discovered that only M2 macrophages (tumor-associated macrophages) present in the TME express higher levels of CCL22/CCL17 than other macrophage subsets (**Figure 2A**). **Figure S2** shows the validation of macrophage subtype characterization. It was also clear that core breast tumor tissue showed higher levels of CCL22/CCL17 transcripts than neighboring non-tumor/normal tissue (**Figure 2A**). We used correlation coefficient plots to show the relationship between CCR4 and CCL22/CCL17 expression in core breast tumor tissue, which revealed a strong positive connection between CCR4 and CCL22/CCL17 in breast tumor tissue (**Figure 2A**, lower panel). The ability of CCR4^+^ Tregs to migrate towards the CCL22 chemokine gradient was assessed using a trans-well migration assay in which Tregs from breast cancer patient blood were placed at the top of the chamber and a gradient of recombinant CCL22 was adjusted in the lower chamber containing cell culture media. It was observed that as the concentration of CCL22 increased, the percentage of migrating CCR4^+^ Tregs increased (**Figure 2B**). In the lower panel, a graphical representation of the same has been provided (**Figure 2B**). CCL22 and CCL17 levels in breast cancer tissue are associated with strong Treg infiltration and consequent immunosuppression; hence, it could be used as a prognostic marker. Several studies have also found that Treg cells dampen the immune system by producing the immunosuppressive cytokine IL10 (12). As shown in **Figure 2C**, the alteration of CCR4 expression in Treg has no negative impact on its immunosuppressive function since the level of intracellular IL10 is not affected. Furthermore, the Annexin-V positivity test revealed that CCR4 expression does not confer a survival advantage to Tregs. All of this suggests that the chemokine receptor CCR4 is exclusively involved in the tumor-specific migration of Tregs without affecting its survival or suppressive activities.

### FOXP3 Binds at the *CCR4* Promoter and Regulates CCR4 Expression in Treg Cells

**Figure 3:**
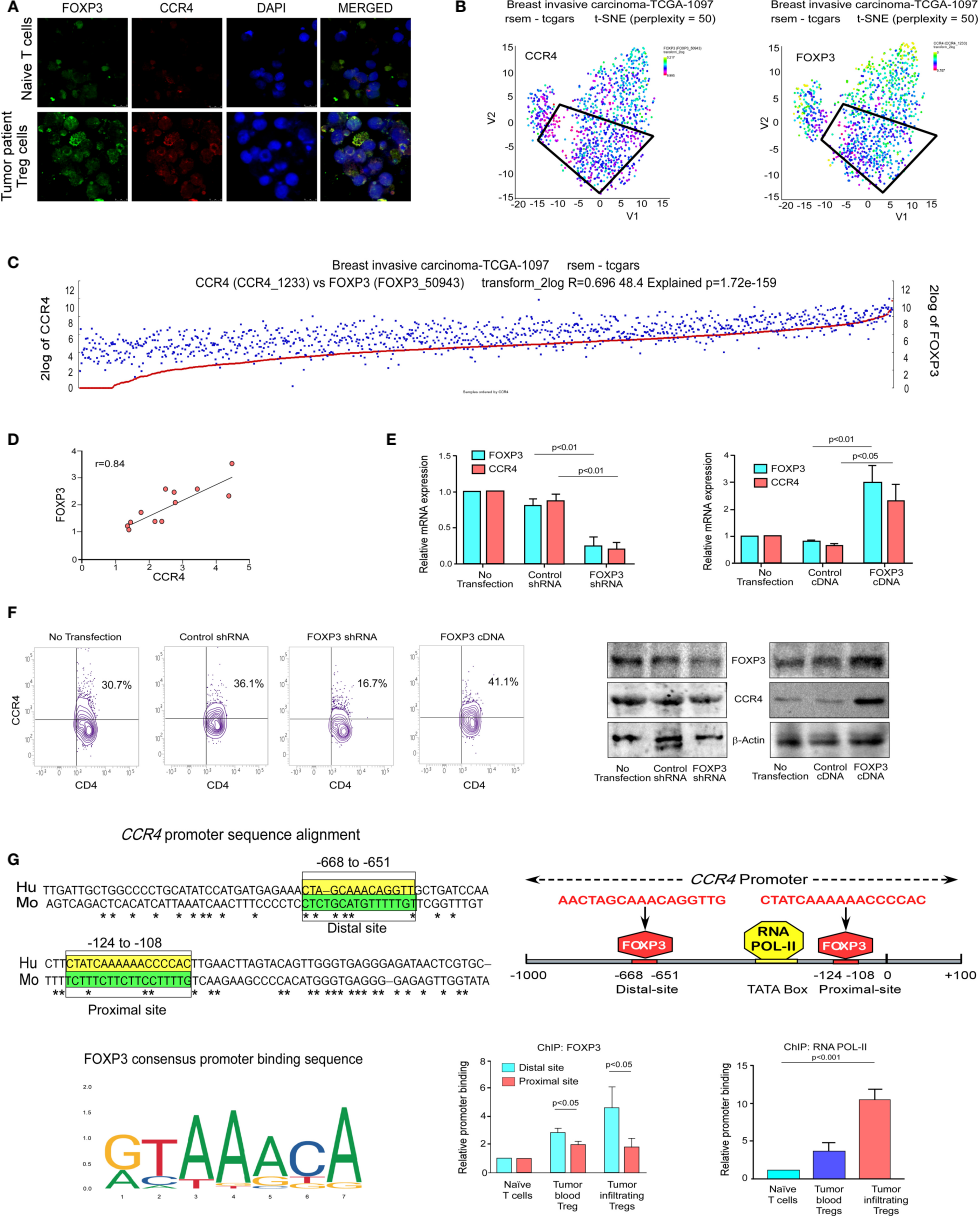
FOXP3 binds at the *CCR4* promoter and regulates CCR4 expression in Treg cells. **(A)** The confocal microscopy data showing the expression of CCR4 and FOXP3 in naïve T cells and Treg cells of breast cancer patients. **(B)** the t-SNE plot of TCGA data from invasive breast carcinoma patients (*n* = 1,097), revealing CCR4 and FOXP3 expression levels. **(C)** FOXP3 and CCR4 correlation plot were derived from TCGA data of invasive breast cancer patients (*n* = 1,097). **(D)** Representative correlation coefficient plots depicting the relationship between CCR4 and FOXP3 in core breast tumor tissue taken from breast cancer patients at various stages (*n* = 12). **(E)** CCR4 and FOXP3 relative mRNA expression in FOXP3-ablated (left panel) and FOXP3-overexpressed (right panel) Tregs induced by shRNA or cDNA. **(F)** CCR4 protein expression in FOXP3-deficient and FOXP3-overexpressed Tregs (flow cytometry; left panel, Western blot; right panel). **(G)** The Fork-head DNA-binding site has been identified in a Clustal W depiction of multiple sequence alignment of the *CCR4* promoter between human and mouse (upper left panel). The consensus DNA-binding sequence for fork-heads has been discovered (obtained from Jasper) (lower left panel). On the *CCR4* promoter, there are two FOXP3-binding sites (Distal site: −668 bp to −651 bp and Proximal site: −124 bp to −108 bp) as well as an RNA POL-II binding region (TATA-Box) (upper right panel). The relative binding of FOXP3 at the *CCR4* promoter’s distal (−668 bp to −651 bp) and proximal (−124 bp to −108 bp) sites are depicted graphically (lower middle panel). The relative binding of RNA POL-II to the TATA-box of the *CCR4* promoter has been graphically depicted (bottom right panel). GAPDH and β-actin were used as internal controls. The values are the mean ± SD of three sets of independent experiments.

We decided to investigate FOXP3’s activity as a transcriptional activator for *CCR4* because it plays such an important role in Treg development and function (37). Being a transcription factor, FOXP3 binds to the promoter region of its target genes. Because FOXP3 nuclear localization is critical for Treg function, we decided to investigate its nuclear translocation. Our findings show that nuclear localization of FOXP3 is more common in tumor patients’ lymphocytes than in normal lymphocytes (**Figure 3A**), implying that it plays a dynamic role as a transcription factor. Our t-SNE study of TCGA data from patients with breast invasive cancer reveals that high CCR4 expression in tumor tissue is linked to high FOXP3 expression (**Figures 3B, C**). With this in mind, we aimed to confirm the positive association between CCR4 and FOXP3 in other cohorts of breast cancer patients. To demonstrate the association between CCR4 and FOXP3 expression in core breast tumor tissue, we created a correlation coefficient plot, which further indicates that CCR4 expression is highly correlated with FOXP3 expression in breast tissue taken from different cohorts (**Figure 3D**).

We ablated or overexpressed FOXP3 in Treg cells and grew them on a breast tumor organoid bed to replicate the *ex vivo* TME to better understand the role of FOXP3 in *CCR4* transcriptional activation. Flow cytometry was used to confirm Treg production *ex vivo* (**Figure S1**). CCR4-mRNA expression is very low in FOXP3-ablated Tregs and very high in FOXP3-overexpressed Tregs, as seen in **Figure 3E**. After FOXP3 depletion/overexpression, CCR4 expression at the protein level as well as at the cell surface showed a consistent pattern with mRNA levels (**Figure 3F**). As a transcription factor, FOXP3 binds to the regulatory area of its target gene’s promoter, prompting us to investigate FOXP3’s binding site at the *CCR4* promoter. For the same reason, we used *in silico* analysis to examine the *CCR4* promoter and observed two FOXP3-binding sites at the −668 bp to −651 bp (distal site) and −124 bp to −108 bp (proximal site) regions of the promoter. **Figure 3G** shows the FOXP3 (DNA-binding domain) consensus promoter binding sequence (obtained from Jasper). This sequence is found in the regulatory region of all FOXP3 target genes; the *CCR4* promoter contains the same DNA-binding region, prompting us to investigate FOXP3’s binding to the *CCR4* promoter. The *in silico* finding was validated by ChIP analysis. FOXP3 binding at the distal site was stronger than at the proximal position (**Figure 3G**), showing that the distal region is the most critical FOXP3-binding site in the *CCR4* promoter. We also examined RNA POL-II binding on the *CCR4* promoter to confirm that the promoter region was open for FOXP3 binding, and the ChIP signal for RNA POL-II was highest in this region (**Figure 3G**).

### The FOXP3/HAT1 Axis Epigenetically Alters *CCR4* Promoter to Promote Treg Infiltration in the Tumor Site

**Figure 4:**
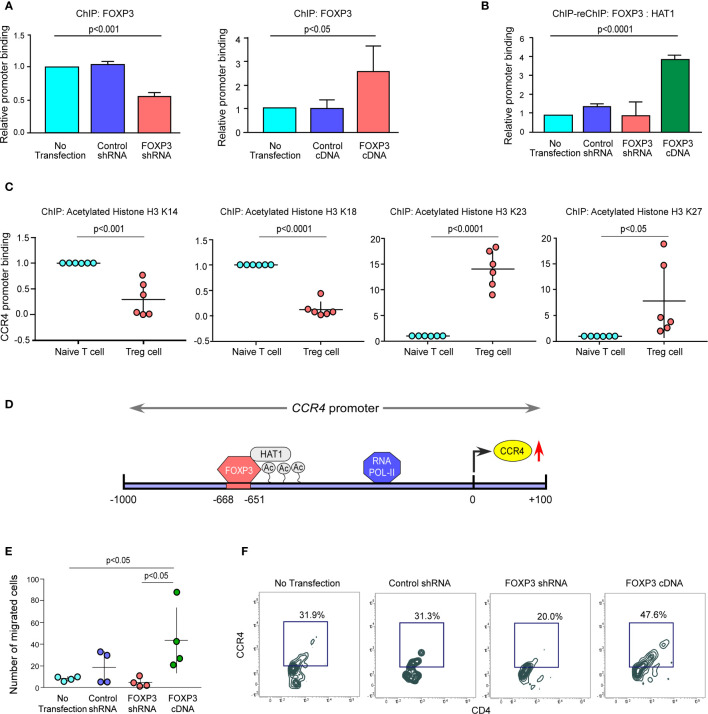
The FOXP3/HAT1 axis epigenetically alters *CCR4* promoter to promote Treg infiltration in tumor site. **(A)** The relative binding of FOXP3 to its putative responsive elements on the *CCR4* promoter has been graphically depicted in FOXP3-ablated/-overexpressed Treg cells. **(B)** The binding of both FOXP3 and HAT1 to the *CCR4* promoter in FOXP3-ablated/-overexpressed Tregs was assessed using a ChIP-Re-ChIP assay, and the results are depicted graphically. **(C)** Relative permissive (K23 and K27) or repressive (K14 and K18) acetylation of histone-3 was determined using a ChIP assay and depicted graphically using site-specific antibodies. **(D)** Schematic illustration of the FOXP3-binding site on the *CCR4* promoter (−668 bp to −651 bp) with HAT1. **(E)** After 6 h, the number of FOXP3-ablated/overexpressed Treg cells in the lower chamber of the trans-well plate that migrated in response to recombinant CCL22 was counted and graphically displayed. **(F)** Flow cytometry was used to determine the percentage of FOXP3-ablated/overexpressed Treg cells that migrated in response to recombinant CCL22 in a trans-well plate (representative plot). The values are the mean ± SD of three sets of independent experiments.

We altered FOXP3 in *ex vivo*-generated Tregs to better understand the processes through which FOXP3 modulates *CCR4* transcription. We found that ablation of FOXP3 decreased the promoter binding, but overexpression of FOXP3 boosted it (**Figure 4A**). Because of the permissive and repressive deacetylation state of histone-3 in naïve T cells, the *CCR4* promoter stays inaccessible. FOXP3 overexpression increased HAT1 binding to the *CCR4* promoter’s distal regions, modifying the chromatin structure and making it accessible to FOXP3 (**Figures 4B, C**). Interestingly, both FOXP3 and HAT1 binding is required for permissive (K23 and K27) or repressive (K14 and K18) acetylation of histone-3 in Tregs to increase CCR4 expression. **Figure 4D** shows a schematic depiction of the FOXP3-binding region on the *CCR4* promoter at the distal region coupled with HAT1. FOXP3 expression in Tregs was ablated or overexpressed by genetic manipulation, and the ability of the altered Tregs to migrate was monitored using a trans-well migration assay in the presence of recombinant CCL22 to confirm our hypothesis that FOXP3 regulates Treg infiltration in the tumor site. The number of migrating cells in the lower chamber was much higher in FOXP3-overexpressed cells than in FOXP3-ablated cells, according to the data in **Figure 4E**. Concurrently, CCR4 expression was found to be higher in the migrating Treg cells (**Figure 4F**). In conclusion, the FOXP3/HAT1 complex induces acetylation of the *CCR4* promoter in tumor Tregs, which regulates Treg infiltration in the TME.

### FOXP3 Regulates CCR4 Expression and Infiltration of Tregs in the Tumor Site in the *In Vivo* Animal Model

**Figure 5:**
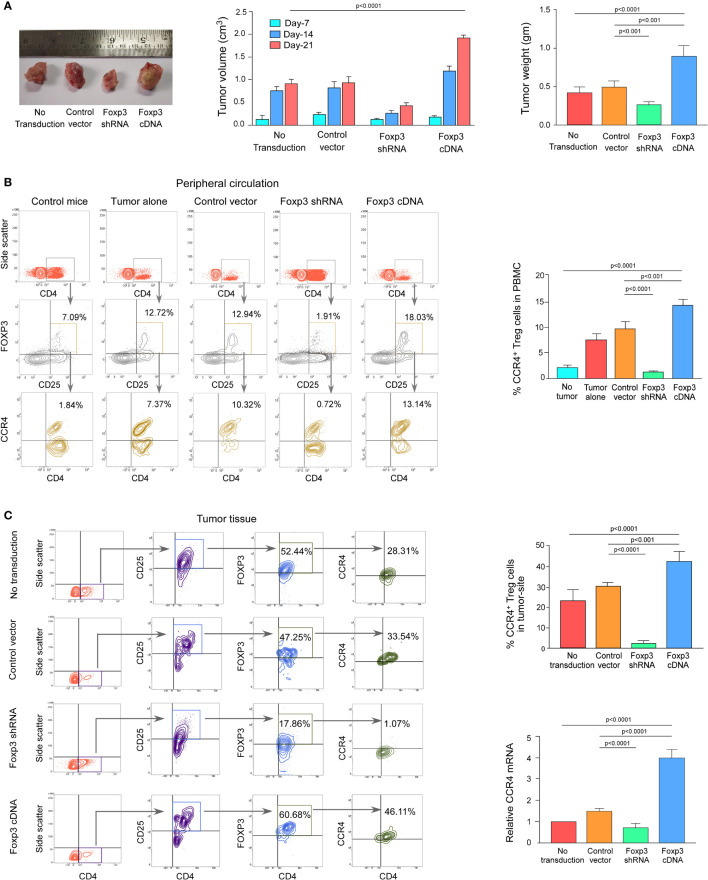
FOXP3 regulates *Ccr4* transcription and infiltration of Tregs in mouse mammary carcinoma site. **(A)** In the tail veins of isogenic mammary cancer (4T1)-transplanted BALB/c mice, lentiviruses expressing Foxp3-shRNA or Foxp3-cDNA were injected (*n* = 6). Mice were sacrificed after 21 days, and the tumor was detached (left panel). The volume and weight of the tumor were measured and graphed (middle panel, right panel). CCR4 and FOXP3 positivity in Treg cells from **(B)** peripheral circulation and **(C)** tumor site were flow cytometrically analyzed (left panels) and graphically plotted (right panels). CCR4-mRNA transcript levels in tumor-infiltrating Tregs were measured using real-time PCR and graphed (lower right panel). As an internal control, GAPDH was used. The values are the mean ± SD of two sets of independent experiments performed in triplicate.

**Figure 6:**
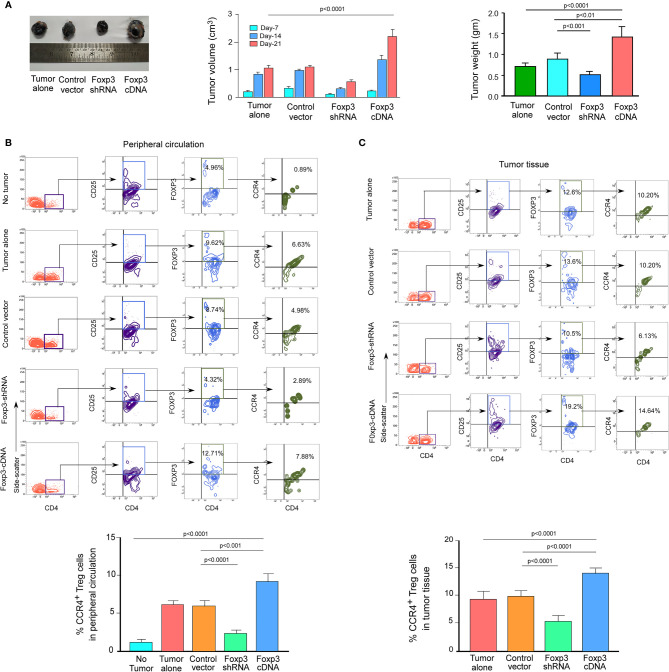
FOXP3 regulates *Ccr4* transcription and infiltration of Tregs in mouse melanoma site. **(A)** Foxp3-shRNA or Foxp3-cDNA lentiviruses were injected into the tail veins of B16/F10 melanoma-transplanted C57/BL6 mice (*n* = 6). Mice were sacrificed after 21 days, and the tumor was detached (left panel). The volume and weight of the tumor were measured and graphed (middle panel, right panel). CCR4 and FOXP3 positivity in Tregs from **(B)** peripheral circulation and **(C)** tumor site were flow cytometrically analyzed (upper panels) and graphically plotted (lower panels). The values are the mean ± SD of two sets of independent experiments performed in triplicate.

We wanted to see if this phenomenon was true in the *in vivo* animal model because both breast cancer patient-derived Tregs and *ex vivo*-generated Treg cells showed a dependency of FOXP3 in the transcriptional regulation of *CCR4*, the key regulator of Treg infiltration in the TME. We employed 4T1-bearing breast carcinoma BALB/c mice and melanoma (B16/F10)-bearing C57/BL6 mice for this study. To genetically manipulate FOXP3 expression in mice, the mice were injected with lentivirus harboring Foxp3-shRNA or Foxp3-cDNA clones. The mice were monitored for 21 days after treatment. In both the breast carcinoma (**Figure 5A**) and melanoma (**Figure 6A**) models, FOXP3 ablation significantly reduced tumor volume and mass. The decrease in tumor size is accompanied by a decrease in CCR4^+^ Treg generation both in the peripheral circulation (**Figures 5B**, **6B**) and at the tumor site (**Figures 5C**, **6C**), demonstrating that FOXP3 transcriptionally activates *CCR4* in both *in vitro* and *in vivo* settings. FOXP3 overexpression, on the other hand, enhanced tumor size (**Figures 5A**, **6A**) by increasing the percentage of CCR4^+^ Tregs in the peripheral circulation (**Figures 5B**, **6B**) as well as at the tumor site (**Figures 5C**, **6C**).

## Discussion

Immune systems are essential for detecting cancer cells and activating efficient immune responses to eliminate them (43). Immune escape is a major hallmark of cancer growth, as it transforms from immunological surveillance (tumor eradication) to immune tolerance (tumor progression) (44). Researchers have long sought to manipulate immune cells to improve the efficacy of immune responses. Cancer immunotherapy involving the suppression of critical Treg-specific proteins like CD25 and the blockade of immune-checkpoint molecules like CTLA4 and PD1 has demonstrated great clinical results in a variety of cancers (45, 46). Long-term survival was observed in approximately 20% of patients treated with immune checkpoint inhibitors in a collective meta-analysis, whereas a large proportion of patients (nearly 80%) experience disease relapse after treatment, highlighting the role of Tregs as a stumbling block to successful immunotherapy. However, excessive reduction of the global pool of Tregs without cause can put the host system at risk of autoimmunity. As a result, detecting tumor-infiltrating Tregs and controlling them has become increasingly critical for effective immunotherapy that also reduces the danger of autoimmunity. Several preclinical and clinical investigations demonstrate that Treg infiltration in the TME interferes with treatments, prevents tumor-bearing hosts from developing antitumor immunity, and promotes tumor progression. As a result, contemporary cancer immunotherapy research has focused on preventing Tregs from infiltrating the TME to increase anti-tumor immunity (47).

Emerging evidence reveals that Treg compartmentalization and trafficking are tissue-specific, and that different chemokine receptors may play a role in Treg trafficking at specific tissue sites (24). Because CCR4 is a tumor-specific chemokine receptor, we examined its expression in Tregs from both the peripheral circulation (source) and the tumor site (destination) in breast cancer patients. CCR4^+^ Tregs have a higher frequency distribution in the tumor site, but CCR4^-^ Tregs dominate in the peripheral circulation, implying that Tregs that gain CCR4 expression in the peripheral circulation travel to the tumor site. This infiltrating CCR4^+^ Treg expands in the tumor site, mounting the tumor immune escape. It is noteworthy that CCL22^hi^/CCL17^hi^ breast tumor tissues are likewise CCR4^hi^. In the TME, the core tumor cells as well as tumor-associated macrophages secrete high amounts of CCL22/CCL17. Finally, the CCR4-CCL22/CCL17 axis increases the accumulation of IL10-producing immunosuppressive Tregs in the TME.

Anti-CCR4 mAb could be an excellent therapy option for individuals with CCR4^+^ neoplasms, as well as a novel strategy for treating cancers including HL, B-CLL, ovarian cancer, and EBV-associated disease, in which CCR4^+^ Tregs prevent the host immune response to the tumor or virus-infected cells (48). Mogamulizumab is a humanized monoclonal antibody that targets CCR4 (36). It has been evaluated in humans for the treatment of CCR4^+^ adult T cell leukemia/lymphoma that has relapsed or become refractory. This humanized antibody is also being looked into as a possible treatment for HTLV1–associated myelopathy (49). CCR4 is a promising target for antibody-based immunotherapy in cutaneous T-cell lymphoma (CTCL) and Tregs because of its high expression. In HL-bearing humanized mice, chimeric defucosylated anti-CCR4 mAb (KM2760) dramatically boosted the number of tumor-infiltrating NK cells that mediate ADCC and decreased the number of tumor-infiltrating FOXP3-positive Treg cells (50). The terminally differentiated and most suppressive effector Treg cells predominantly express CCR4 in both cancer tissues and peripheral blood and anti-CCR4 mAb treatment selectively depletes effector Treg cells and induces anti-tumor immunity (35). Anti-CCR4 mAb could be an appropriate treatment for a variety of malignancies, not only because it kills CCR4-expressing tumor cells directly, but also because it overcomes Treg cells’ suppressive influence on the host immune response.

Despite the importance of CCR4 in Treg tumor invasion, there is very little information on its transcriptional regulation in tumor-associated Treg cells. However, few studies provide light on the transcriptional regulation of this chemokine receptor in different cancer types. For example, CCR4 is controlled by NFκB in colorectal cancer, promoting its metastasis (51). Fra-2 enhances CCR4 expression in adult T-cell leukemia (52), and HTLV-1 viral factor-generated GATA3 stimulates CCR4 expression in CD4^+^ T cells (53). All of these studies have revealed that lineage-specific transcription factors play a critical role in the regulation of CCR4 expression.

FOXP3 carries out its diverse functions by regulating the transcription of its target genes. The t-SNE analysis of TCGA data from patients with breast invasive cancer also revealed a strong connection between CCR4 and FOXP3, which was further validated in our laboratory. Interestingly, it was observed that both human and murine *CCR4* promoters have consensus FOXP3-binding regions, and in naïve T cells, both the sites remain inaccessible due to their highly condensed heterochromatin state. FOXP3 recruits HAT1 at the distal FOXP3-binding site of *CCR4* promoter to convert highly condensed heterochromatin state to an open euchromatin state by permissive acetylation of lysine residues of histone-3. As a result of this epigenetic landscape, FOXP3 and RNA POL-II get access at the distal-promoter region to transcribe *CCR4*.

In breast cancer patients, a high amount of TGFβ released by tumor cells, combined with sustained TCR-stimulation, increases the acquired expression of FOXP3 in CD4^+^CD25^-^ T cells, allowing them to develop into Treg cells. TGF/TCR signaling also prevents DNA methyltransferase-1 from binding to the FOXP3 promoter, allowing FOXP3 expression to be preserved in Tregs (54). We also established that TGFβ-induced FOXP3 enhances *CCR4* transcription in Treg cells, making them amenable for tumor site infiltration (**Figure S3**). In both mammary carcinoma- and melanoma-bearing mice, ablation of FOXP3 reduces the generation of CCR4^+^ Tregs and hence their infiltration into the tumor site. Reduced Treg infiltration resulted in an increase in anti-tumor immunity, which resulted in a reduction in tumor growth. The translational *in vivo* tumor model suggested that altering the expression of either CCR4 or FOXP3 in cancer patients could improve the efficacy of chemotherapy or immunotherapy.

Nowadays, the primary goal of cancer therapy is to limit tumor growth and increase treatment efficacy. The body’s innate immune system works by recognizing and attacking “foreign” cells, but cancer cells disguise themselves to avoid detection by the host immune system. Tregs play a role in this scenario by suppressing both T-effector and T-cytotoxic cells, effectively making tumor cells “friendly.” Immunotherapy’s purpose is to help the host immune system recognize cancer cells as invaders and wage war on them, which can be done even more successfully by focusing on tumor cells’ “friends”. The tolerogenic environment of a cancer patient is created by Treg infiltration in the TME. When the balance between the proliferation and function of Treg and Teff cells is broken, tumor cells multiply more quickly.

**Figure 7:**
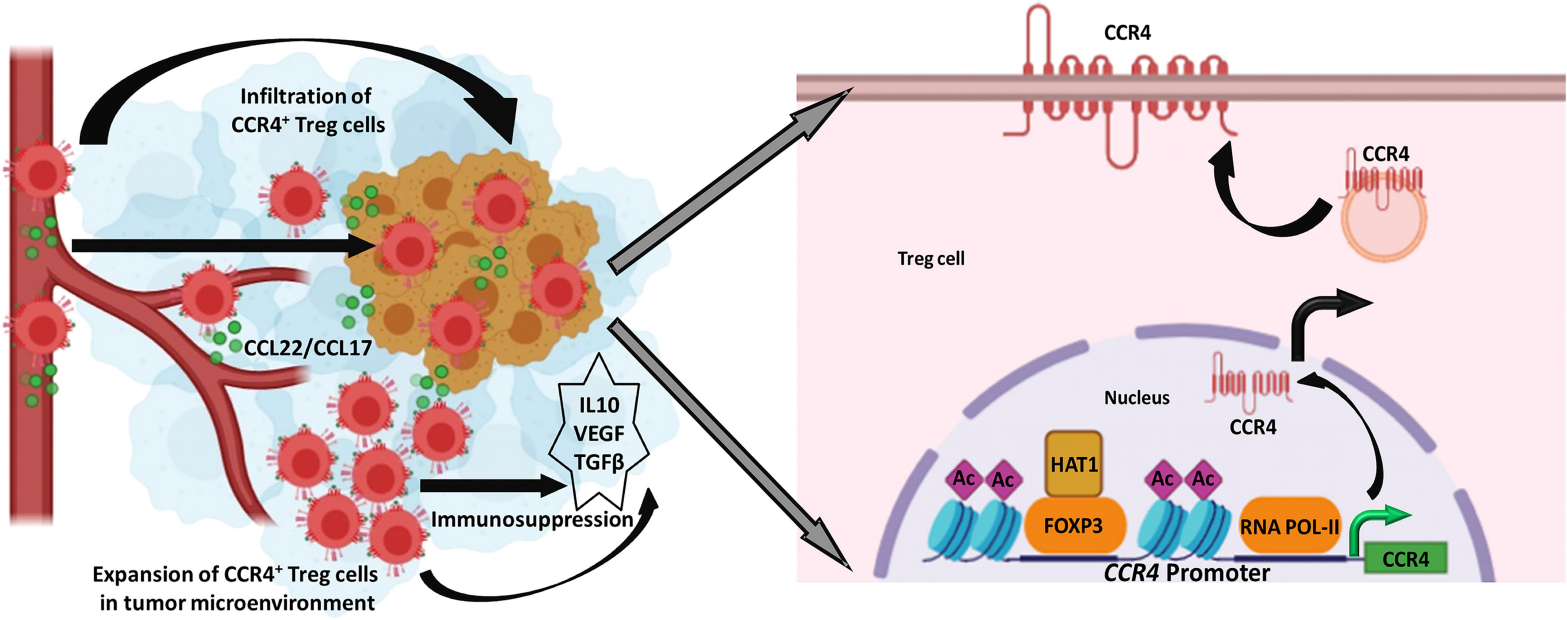
FOXP3-transcribed CCR4 expression regulates Treg infiltration in tumor site. Left: CCR4^+^ Tregs are infiltrated in the tumor microenvironment in response to tumor-secreted CCL22/CCL17 to promote tumor immune escape. Right: FOXP3 in the association of HAT1 binds to *CCR4* promoter to epigenetically modify it for optimal transcription of *CCR4* gene.

Treg levels in the TME are linked to a cancer patient’s poor prognosis and distant metastases. As a result, our research reveals that Treg infiltration in the TME is one of the major roadblocks to building anti-tumor immune escape. We reasoned that reducing CCR4 expression could aid in controlling these immunosuppressive pro-tumor Tregs and hence slow tumor growth. As a result, Treg infiltration interference in the TME has become a significant focus of research in recent years. Despite this, none of this research described how CCR4 expression in Treg cells is controlled. Our findings revealed a novel role for FOXP3 in the regulation of CCR4 expression in tumor-infiltrating Tregs (**Figure 7**) and gave a molecular insight as to how immunosuppressive Tregs infiltrate into the TME, allowing us to disrupt the pathway implicated and increase cancer immunity.

## Data Availability Statement

The original contributions presented in the study are included in the article/**Supplementary Material**. Further inquiries can be directed to the corresponding author.

## Ethics Statement

The studies involving human participants were reviewed and approved by Ethics committee, ESI Post Graduate Institute of Medical Science and Research, Kolkata, India, the Institute of Post-Graduate Medical Education and Research Oversight Committee, and Human Ethics Committee, Bose Institute. The patients/participants provided their written informed consent to participate in this study. The animal study was reviewed and approved by Bose Institute Ethics Committee.

## Author Contributions

GS conceptualized and supervised the study and edited the manuscript. TS, SD, DC, SP, SB, AP, UB, SC, SM, and AG performed experiments and analyzed data. TS and SD wrote manuscript draft. DS provided support with clinical samples. SP and SB helped in bioinformatics data analysis. KJ provided support with animal experiments. All authors contributed to the article and approved the submitted version.

## Funding

The study was funded by grants from Department of Biotechnology, Government of India.

## Conflict of Interest

The authors declare that the research was conducted in the absence of any commercial or financial relationships that could be construed as a potential conflict of interest.

## Publisher’s Note

All claims expressed in this article are solely those of the authors and do not necessarily represent those of their affiliated organizations, or those of the publisher, the editors and the reviewers. Any product that may be evaluated in this article, or claim that may be made by its manufacturer, is not guaranteed or endorsed by the publisher.
