# Supplementary material for: FOXP3/HAT1 Axis Controls Treg Infiltration in the Tumor Microenvironment by Inducing CCR4 Expression in Breast Cancer
Source: Front Immunol. 2022 Feb 9;13:740588. doi: 10.3389/fimmu.2022.740588 (PMC8863663; doi:10.3389/fimmu.2022.740588)
Supplement: Supplementary file 1 [file DataSheet_1.pdf]

Supplementary Materials:

FOXP3/HAT1 axis controls Treg infiltration in the tumor microenvironment by inducing CCR4 expression in breast cancer

Tania Sarkar, Subhanki Dhar, Dwaipayan Chakraborty, Subhadip Pati, Sayantan Bose, Abir K Panda, Udit Basak, Sourio Chakraborty, Sumon Mukherjee, Aharna Guin, Kuladip Jana, Diptendra K. Sarkar, Gaurisankar Sa

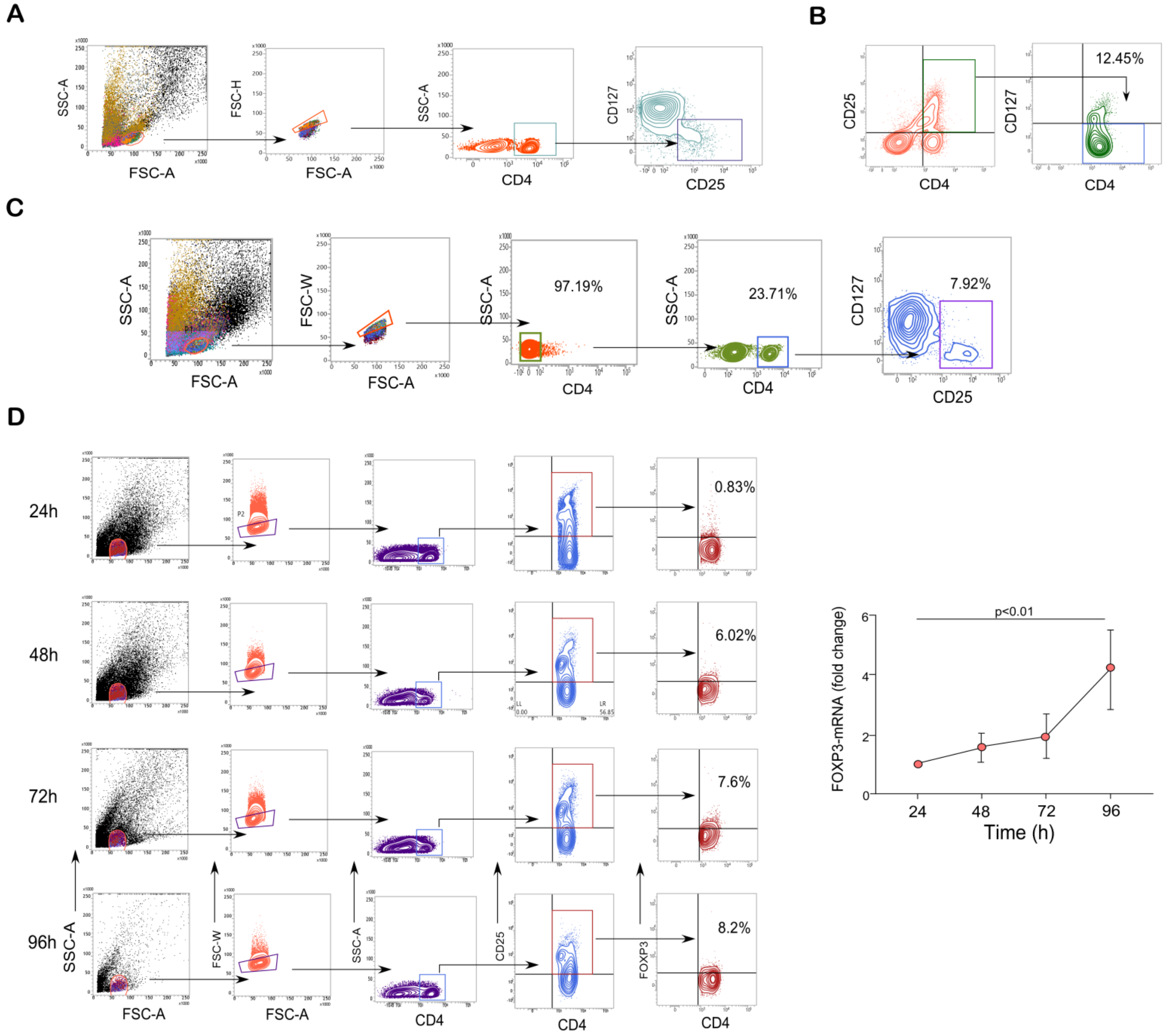

Figure-S1. Gating strategy for sorting of naïve T cell and Treg cells and ex-vivo generation of Tregs: (A) Gating strategy for isolation of $CD4^{+}CD25^{+}CD127^{-}$ Treg cells. **(B)** Flow-cytometric representation of the *ex-vivo* generated $CD4^{+}CD25^{+}CD127^{-}$ Treg cells in tumor tissue bed after 72h. **(C)** Flow-cytometric representation of the exclusion of the doublets and the validation of the cell viability via 7AAD exclusion method. **(D)** Flow-cytometric representation of the *ex-vivo* generated $CD4^{+}CD25^{+}FOXP3^{+}$ Treg cells in a time-dependent manner (left-panel). Graphical representation of the relative FOXP3 mRNA expression in the *ex-vivo* generated $CD4^{+}CD25^{+}FOXP3^{+}$ Treg cells in a time-dependent manner (right-panel). GAPDH was used as internal control. Values are mean ±SD or representatives of three sets of independent experiments.

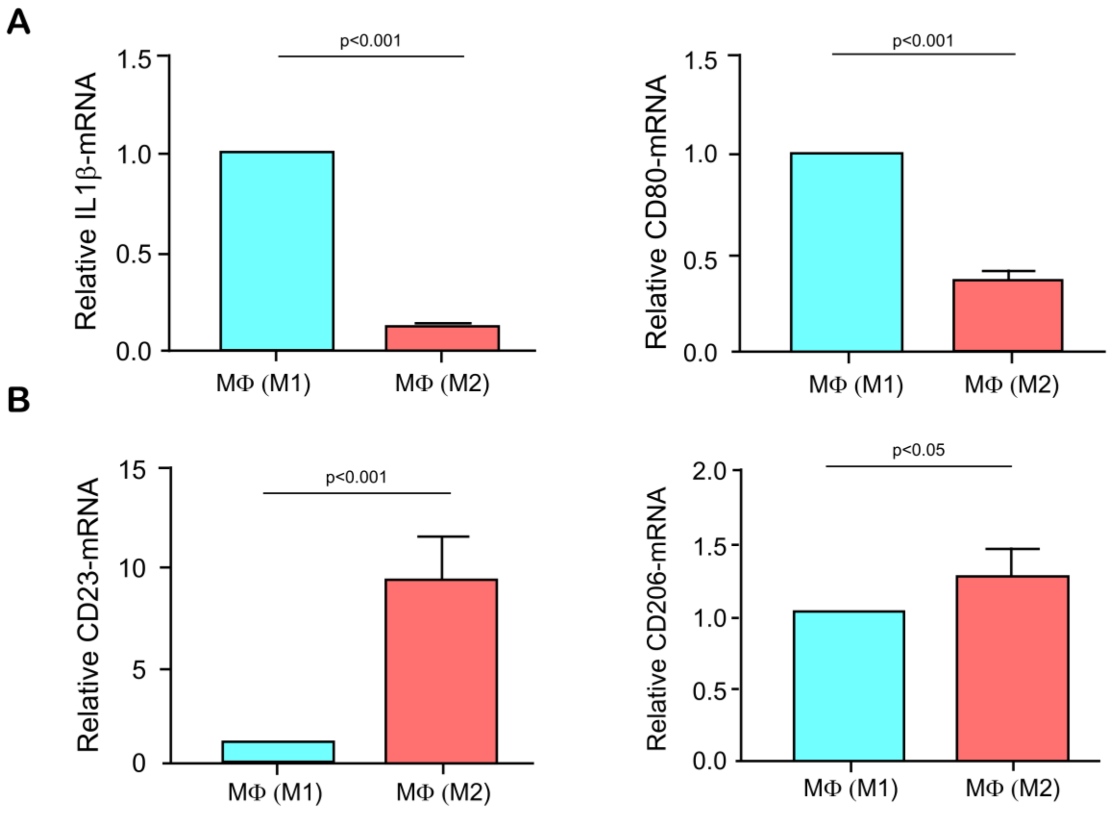

Figure-S2. Characterization of Mϕ-M1 and Mϕ-M2: (A) Relative mRNA expression of IL1β (left-panel) and CD80 (right-panel) in different macrophages subpopulations (M0, M1 and M2). **(B)** Relative mRNA expression of CD23 (left-panel) and CD206 (right-panel) in different macrophages subpopulations (M0, M1 and M2). GAPDH was used as internal control. Values are mean ±SD or representatives of three sets of independent experiments.

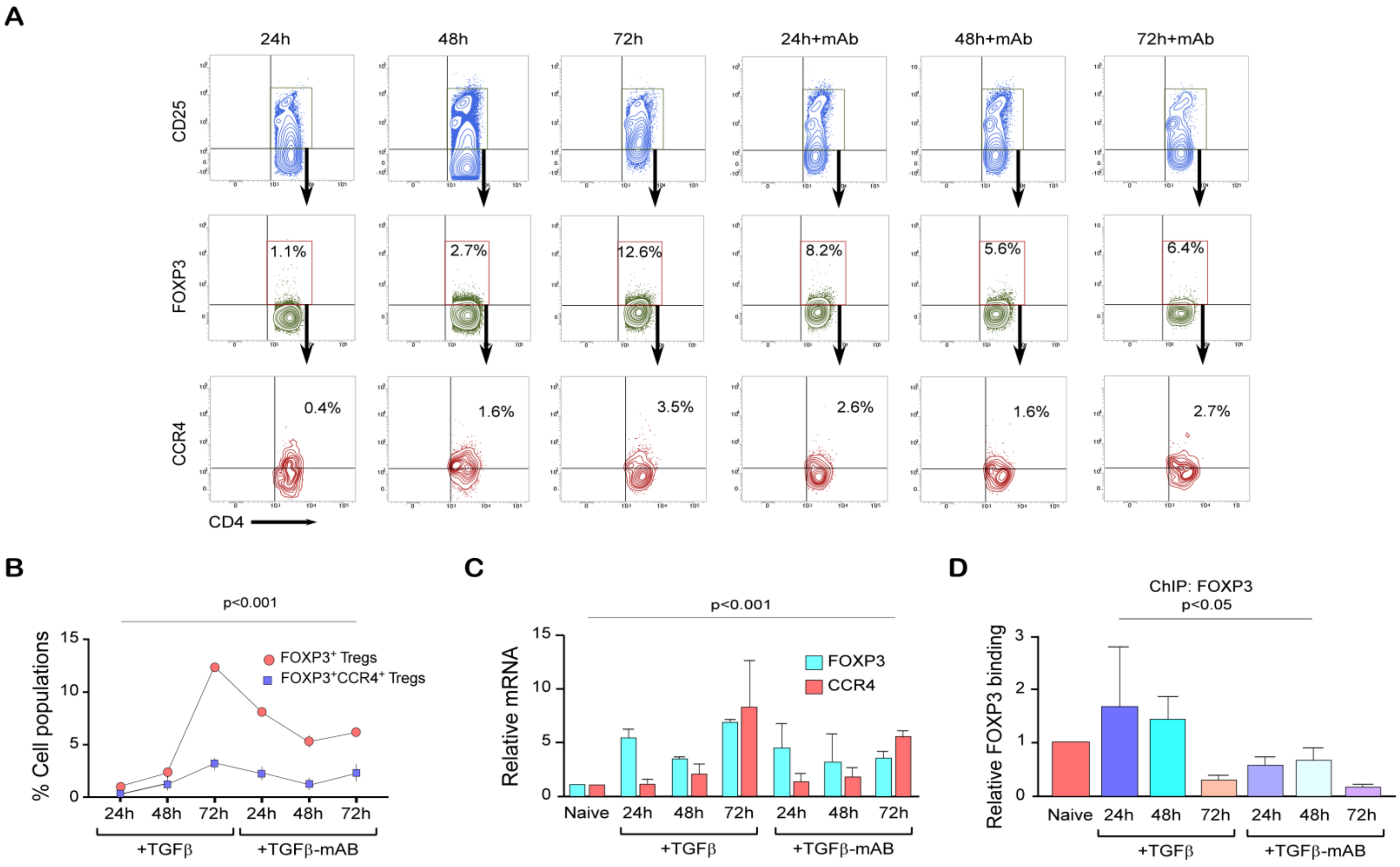

Figure-S3. TGFβ-induced FOXP3 regulates CCR4 expression: (A) Flow-cytometric representation of TGFβ-induced *in-vitro* generation of $CD4^+CD25^+FOXP3^+CCR4^+$ Treg cells from naïve T cells at different time-intervals. **(B)** The flow-cytometric data were graphically represented. **(C)** The relative expression levels of CCR4-/FOXP3-mRNA were analyzed by qPCR in presence of TGFβ. **(D)** Relative binding of FOXP3 on *CCR4*-promoter in presence of TGFβ was analyzed by ChIP assay. GAPDH was used as internal control. Values are mean ±SD or representatives of three sets of independent experiments.

Table-S1: Clinical information of the breast cancer samples used in this study

Serial No.	Age	Size	TNM staging	Grade	ER/PR	Her2
1	60	4cm	T2N1M0	IIB	Positive	Positive
2	42	3cm	T2N0M0	IIA	Negative	Positive
3	50	3cm	T2N0M0	IIA	Positive	Negative
4	32	5cm	T2N0M0	IIA	Positive	Positive
5	40	4cm	T2N1M0	IIB	Negative	Negative
6	31	3cm	T2N0M0	IIA	Positive	Positive
7	57	4cm	T2N1M0	IIB	Negative	Negative
8	59	3cm	T2N1M0	IIB	Positive	Positive
9	34	5cm	T3N1M0	IIIA	Negative	Positive
10	46	6cm	T3N1M0	IIIA	Positive	Negative
11	55	7cm	T3N2M0	IIIA	Negative	Negative
12	54	4cm	T3N2M0	IIIA	Positive	Positive
13	34	6cm	T3N1M0	IIIA	Negative	Positive
14	57	5cm	T3N0M0	IIIA	Positive	Negative
15	65	7cm	T4N1M0	IIIB	Negative	Negative
16	32	7cm	T4N1M0	IIIB	Negative	Positive
17	63	9cm	T4N0M0	IIIB	Positive	Positive
18	43	<2cm	T1N1M0	IB	Positive	Negative
19	38	<2cm	T1N1M0	IB	Positive	Negative
20	52	<2cm	T1N1M0	IB	Positive	Positive
21	42	<2cm	T1N0M0	IA	Positive	Positive
22	54	<2cm	T1N1M0	IB	Positive	Negative
23	58	<2cm	T1N0M0	IA	Positive	Positive

Table-S2: Primers used in the study

CCR4 Human Forward	CTCTGGCTTTTGTTCACTGC
CCR4 Human Reverse	GTGGACTGCGTGTAAGATGA
CCR4 Mouse Forward	GTAGAAGAAGCTCGTCGGTG
CCR4 Mouse Reverse	CTCACAAGAGCATTTCAGAGC
FOXP3 Human Forward	TCATCCGCTGGGCCATCCTG
FOXP3 Human Reverse	GTGGAAACCTCACTTCTTGGTC
FOXP3 Mouse Forward	CCTGGTTGTGAGAAGGTCTTCG
FOXP3 Mouse Reverse	TGCTCCAGAGACTGCACCACTT
GAPDH Human Forward	CCTGCACCACCAACTGCTTA
GAPDH Human Reverse	GGCCATCCACAGTCTTCTGGG
GAPDH Mouse Forward	CATCACTGCCACCCAGAAGACTC
GAPDH Mouse Reverse	ATGCCAGTGAGCTTCCCGTTCAG
CD206 Human Forward	TGGTGGAAGAAGAAGCAGTC
CD206 Human Reverse	TGCTGGAGGATTAGTCAAGG
IL1B Human Forward	GTCTACACCAATGCCCAACT
IL1B Human Reverse	CGGGCTTTAAGTGAGTAGGA
CD80 Human Forward	GGAAAGACATAGCCAACTGC
CD80 Human Reverse	GCTTAGCTGCCATGAGATGT
CD23 Human Forward	ACATCTCCCGCTCCTCTATG
CD23 Human Reverse	GTGCTGTTGGGGTGTACTCT
CCL22 Human Forward	GACTCCTGTACCTTTCTTCGTT
CCL22 Human Reverse	CCCCCTCTCACAGTTCCTAAA
CCL17 Human Forward	CCATTCCCCTTAGAAAGCTGA
CCL17 Human Reverse	ACTTTAATCTGGGCCCTTTGT
Ki67 Human Forward	TCCTTTGGTGGGCACCTAAGACCTG
Ki67 Human Reverse	TGATGGTTGAGGCTGTTCCTTGATG
CCR4 promoter Forward (FOXP3 binding site/distal-site)	CTTCTCACCCTGGCCTCTC
CCR4 promoter Reverse (FOXP3 binding site/distal-site)	GCAATCCTCCCACCTCAGC
CCR4 promoter Forward (RNA POL II-binding site, FOXP3	CTTGGCCTTCGGTCTTGGG

binding site/proximal-site)	
CCR4 promoter Reverse (RNA POL II-binding site, FOXP3 binding site/proximal-site)	GGGTGCGTGGCTTCCTGTG
